# Clinical Impact of Nivolumab in Sinonasal Mucosal Melanoma: A 14-Year Single-Center Retrospective Study and Comprehensive Literature Review

**DOI:** 10.3390/cancers18071174

**Published:** 2026-04-06

**Authors:** Kosuke Terazawa, Ryo Utakata, Ryota Iinuma, Masashi Kuroki, Tatsuhiko Yamada, Hiromasa Ishihara, Ryo Kawaura, Hiroshi Okuda, Kenichi Mori, Hirofumi Shibata, Natsuko Obara, Miki Umeda, Ryoukichi Ikeda, Ken Saijo, Takenori Ogawa

**Affiliations:** 1 Department of Otolaryngology-Head and Neck Surgery, Graduate School of Medicine, Gifu University, Gifu 501-1194, Japan; terazawa.kosuke.y4@f.gifu-u.ac.jp (K.T.); utakata.ryo.y2@s.gifu-u.ac.jp (R.U.); iinuma.ryota.h1@f.gifu-u.ac.jp (R.I.); kuroki.masashi.x3@s.gifu-u.ac.jp (M.K.); yamada.tatsuhiko.a2@f.gifu-u.ac.jp (T.Y.); ishihara.hiromasa.n4@f.gifu-u.ac.jp (H.I.); kawaura.ryo.c9@f.gifu-u.ac.jp (R.K.); okuda.hiroshi.z1@f.gifu-u.ac.jp (H.O.); mori.kenichi.d6@f.gifu-u.ac.jp (K.M.); obara.natsuko.m6@f.gifu-u.ac.jp (N.O.); kanda.miki.b8@s.gifu-u.ac.jp (M.U.); ikeda.ryoukichi.h3@f.gifu-u.ac.jp (R.I.); 2 Department of Otorhinolaryngology-Head and Neck Surgery, Graduate School of Medicine, Mie University, Tsu 514-8507, Japan; hirofumi-shibata@med.mie-u.ac.jp; 3 Department of Clinical Oncology, Graduate School of Medicine, Tohoku University, Sendai 980-8575, Japan; ken.saijo.d6@tohoku.ac.jp

**Keywords:** head and neck cancer, sinonasal mucosal melanoma, immune checkpoint inhibitor

## Abstract

**Background**: Sinonasal mucosal melanoma (SNMM) is a rare aggressive malignancy often resected with postoperative irradiation, with some evidence supporting the use of immune checkpoint inhibitors (ICIs). This study explores the potential efficacy and safety of nivolumab in 16 patients with SNMM, and reviews the literature of ICI use in it. **Methods**: We retrospectively analyzed 16 patients who had pathologically confirmed SNMM treated at Gifu University Hospital over a 14-year period between 2010 and 2024, of whom 11 received nivolumab. Clinical characteristics, treatment modalities, immune-related adverse events (irAEs), progression-free survival (PFS), and overall survival (OS) were examined. Kaplan–Meier and log-rank tests were used for survival analysis. A comprehensive literature review was done regarding ICI in SNMM. **Results**: Among the 16 patients, 11 received nivolumab and five did not. The nivolumab group showed longer median survival (median OS 26 mo vs. 8 mo; *p* = 0.00056) and a median PFS of 13 mo vs. 3 mo, *p* = 0.00175. In recurrent cases, nivolumab was associated with longer median OS (23 mo vs. 9 mo, *p* = 0.015) and PFS (11 mo vs. 3 mo, *p* = 0.019). irAEs occurred in four out of eleven of these cases, leading to nivolumab discontinuation; however, three of these four patients maintained durable disease control. A literature review identified generally favorable outcomes for ICIs in SNMM, although results were varied due to small sample sizes and heterogeneous treatment settings. **Conclusions**: Nivolumab was associated with longer survival in this small cohort in patients with SNMM, including those who discontinued therapy due to irAEs. Our data supports recent meta-analyses demonstrating a benefit of ICIs in SNMM.

## Simple Summary

Sinonasal mucosal melanoma (SNMM) is a rare aggressive malignancy with limited treatment options. Although checkpoint inhibitors are often used for SNMM, proof of efficacy is hampered by the small size of studies. In this study, we reviewed our single-institution data to evaluate the impact of nivolumab. Our findings suggest that nivolumab may be associated with longer overall survival (OS) and slower disease progression compared to alternatives. Some patients developed immune-related adverse events that required stopping treatment; several showed long-durable response. Our data supports recent meta-analyses.

## 1. Introduction

Sinonasal mucosal melanoma (SNMM) accounts for 0.7–1% of all melanomas and 4–8% of sinonasal malignant tumors [1], impacting 1 in 1.5 million people, usually >65 yo [2]. Among those affected, 94% are pN0 [3], and prognosis is worsened by involvement of the orbit and skull base [4]. The rarity of SNMM precludes clinical trials. A 2024 meta-analysis of 459 SNMM patients reported 28-month median OS, with 1-year, 3-year, and 5-year survival rates of 70%, 40%, and 30% [5]. A National Cancer Database analysis of 1874 patients reported similar 5-year overall survival of 24% [6]. SNMM is resected (if possible), and postoperative radiation therapy (RT) is given. The standard treatment for resectable SNMM is surgery followed by postoperative radiation therapy (PORT). Achieving negative margins is challenging if the tumor invades the orbit and skull base [7]. RT improves local controls by ~50% but has limited OS impact [8]. Chemotherapy regimens for SNMM have frequently included multiple cytotoxic drugs [9]. Regimens such as DAV (DTIC, ACNU, VCR) and high-dose interferon therapy have been used, but response rates have generally been low, and toxicity significant [10]. ICIs have markedly impacted melanoma treatment, showing good responses in advanced or metastatic disease [11].

Nivolumab was approved for insurance coverage for melanoma in 2014 [12]. Later, a second anti-PD-1 antibody (pembrolizumab) was approved, as has the anti-CTLA-4 antibody ipilimumab [13,14]. SNMM has a molecular profile and clinical characteristics which are slightly different from other melanomas. *BRAF* mutations, common in cutaneous melanoma, are found in only 4–8% of SNMM cases, while *KIT* is found in 5–22%. *NRAS* mutations are observed in 14–42% of SNMM cases [15,16], at codon 12–13 (not codon 61 as in cutaneous melanoma) [17]. Another characteristic feature is loss of *PTEN* expression, which is rarely seen in other melanomas [18].

In this study, we contribute by adding to earlier reports regarding the efficacy and safety of ICI for SNMM, and by reviewing the literature of ICI use in SNMM supplement recent meta-analyses.

## 2. Materials and Methods

### 2.1. Patients

This study included patients with SNMM treated at Gifu University Hospital between October 2010 and October 2024. All patients underwent clinical evaluation, including endoscopy and imaging studies, to determine tumor extent and eligibility for surgery or systemic therapy. All patients were pathologically diagnosed based on histological examination of specimens obtained via biopsy or surgery. Nivolumab (Ono Pharmaceutical Co., Ltd., Osaka, Japan) was administered as adjuvant treatment after surgery, or to patients with unresectable disease or distant metastasis at initial diagnosis. Nivolumab was administered at 240 mg every 2 weeks and 480 mg every 4 weeks for stable patients. All relevant clinical data were obtained from patient medical records. This study was conducted in accordance with the Declaration of Helsinki and was approved by the Ethics Committee of the Graduate School of Medicine, Gifu University (Approval No. 2023-253).

### 2.2. Data Collection and Endpoints

Data on sex, age, performance status (PS), alcohol and tobacco use, clinical stage, treatment modalities, occurrence of adverse events, recurrence, and survival were extracted from medical records. Performance status was assessed using the Eastern Cooperative Oncology Group (ECOG) scale. Tumor staging was determined according to the Union for International Cancer Control (UICC) TNM classification for sinonasal carcinoma. Immune-related Adverse Events (irAEs) were assessed according to the Common Terminology Criteria for Adverse Events, v5.0 (CTCAE v5.0). For prognostic assessment, overall survival (OS) and progression-free survival (PFS) were analyzed. OS was defined as the duration from initiation of first-line treatment to the date of death or last follow-up. PFS was defined as the duration from initiation of first-line treatment to the date of disease progression, death, or last follow-up. Comparisons were performed between the nivolumab-treated group and the other treatment group.

### 2.3. Statistical Analysis

We recognize the issue of statistically comparing a group of 11 patients receiving nivolumab (at some point in their treatment, with only 4 receiving it as adjuvant chemotherapy after both surgery and PORT) with a 2nd group of 5 that did not. The two groups are small and not similar. Survival time analysis was performed using the Kaplan–Meier method, and differences between groups were analyzed using the log-rank test. Multivariate analysis with Cox proportional hazards regression was not performed due to the small sample size, but descriptive statistics were used to summarize patient characteristics and treatment outcomes. Continuous variables were summarized as medians and ranges, while categorical variables were reported as frequencies and percentages. All statistical analyses were performed using EZR (version 3.6.3). A *p*-value < 0.05 was considered significant in all analyses.

## 3. Results

### 3.1. Patient Characteristics

This study included 16 patients with SNMM. The median age was 69 years (range: 33–82 years), and six patients had a history of smoking and drinking. Among the 11 patients that received nivolumab, there were five men and six women. Five were stage 1, five were stage 2, and one was stage 3. Among the five who received other treatments, there were two men and three women, and the average stage was slightly more advanced, with one being stage 1, three stage 2, and one stage 4.

### 3.2. Details of Treatment

Details of all 16 patients are shown in Table 1. Nivolumab was used in six patients for recurrent disease: in one as initial definitive treatment, in four as adjuvant therapy after surgery and RT, and in one as adjuvant after RT without surgery. Before nivolumab became eligible for insurance coverage, perioperative chemotherapy mainly consisted of the DAV regimen (DTIC, ACNU, VCR). Eleven recurred. Three patients received best supportive care (BSC), while eight underwent further treatment, with seven receiving ICI including nivolumab. In Case 8, myasthenia gravis had occurred with nivolumab, and pembrolizumab was used for recurrence. Case 14 received a combination of nivolumab and ipilimumab. Of the 16 patients, five are still alive. Four patients who received nivolumab experienced immune-related adverse events (irAEs) and discontinued nivolumab. Adverse events included skin issues in two patients, myasthenia gravis in one patient, and both colitis and hypothyroidism in one patient. All four of these patients received systemic steroids according to established guidelines, and no treatment-related deaths occurred. Among the four patients who discontinued nivolumab, only one experienced disease progression. These results suggest that some patients may maintain durable responses after discontinuation of ICI, as has been reported in other cancers.

### 3.3. Prognosis of SNMM Cases Treated with Nivolumab

We recognize that a limitation of this report is that the two groups are not otherwise similar, and we caution the reader not to overinterpret any apparently statistically significant finding that may be due to factors other than just nivolumab use.

**Figure 1:**
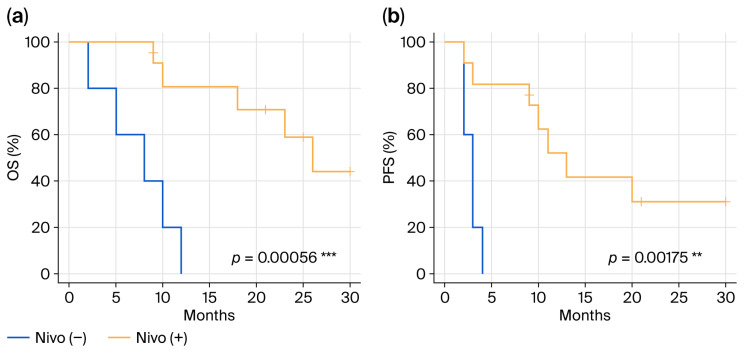
Prognosis of all cases. In all cases, prognosis ((**a**) OS, (**b**) PFS) was compared between the group with nivolumab (*n* = 11) and the group without nivolumab (*n* = 5). Cases receiving nivolumab had significantly longer OS and PFS. OS—overall survival; PFS—progression-free survival. ***; *p* < 0.001, **; *p* < 0.01. These two groups are not otherwise necessarily similar and the reader is cautioned not to overinterpret any “statistical significance”.

First, we compared prognosis (OS, PFS) based solely on nivolumab use. Median OS was 8 months in the five cases without nivolumab (*n* = 5) compared to 26 months in the 11 with nivolumab (*p* = 0.00056) (Figure 1a). Similarly, median PFS was 3 months in the group without nivolumab, compared to 13 months in the group with nivolumab (*p* = 0.00175) (Figure 1b). An additional analysis excluding Case 7, who received BSC without initial treatment, and similarly observed significant prolongation of both OS and PFS (median OS; 9 mo vs. 26 mo, *p* = 0.00184, median PFS; 3 mo vs. 13 mo, *p* = 0.00475).

**Figure 2:**
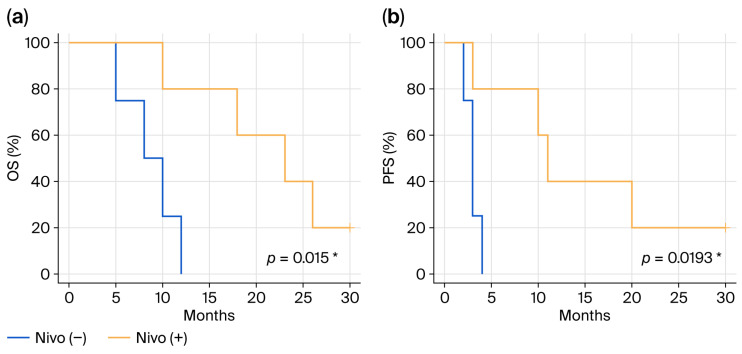
Prognosis of recurrent and metastatic cases. In recurrent and metastatic cases, prognosis ((**a**) OS, (**b**) PFS) was compared between the group with nivolumab (*n* = 5) and the group without nivolumab (*n* = 4). As with the overall cases population, recurrent and metastatic cases treated with nivolumab showed significantly longer OS and PFS. OS—overall survival; PFS—progression-free survival. *; *p* < 0.05.

Next, we compared outcomes in recurrent and metastatic cases between the group with nivolumab (*n* = 5) and the group without nivolumab (*n* = 4). The median OS was 9 months in the group without nivolumab, and 23 months in the group with nivolumab (*p* = 0.015) (Figure 2a). Similarly, the median PFS was 3 months in the group without nivolumab, compared with 11 months in the group with nivolumab (*p* = 0.0193) (Figure 2b). These results suggest that ICIs may be potentially useful as treatment for recurrent and metastatic SNMM, where effective treatments are limited.

## 4. Discussion

In this study, we examined the safety and efficacy of nivolumab in the management of SNNM, adding 11 patients to those reported in recent meta-analyses. As BRAF mutation is harbored in only 8% of SNMM cases, BRAF inhibitors are rarely indicated for treatment [19].

SNMM is a rare malignancy, and most evidence regarding ICI comes from studies on mucosal melanoma as a broader category. Therefore, it is important to consider data from mucosal melanoma when interpreting results for SNMM. Several analyses and systematic reviews have also evaluated the efficacy of ICI in mucosal melanoma. A pooled analysis reported an ORR of 23.3% with nivolumab and 37.1% with nivolumab plus ipilimumab, with median FPS of 3.0 and 5.9 months, respectively [20]. Similarly, an international retrospective cohort study involving more than 500 patients with mucosal melanoma demonstrated clinically meaningful activity of anti-PD-1–based therapy [21].

There are no prospective analyses of therapeutic effects of ICI in SNMM because of their rarity; retrospective studies like this paper typically involve only a few patients. A systematic review of retrospective studies in SNMM reported favorable outcomes with ICI therapy in SNMM [19]. In an international multicenter retrospective study of 505 SNMM patients, ICIs for the management of recurrent or persistent disease were associated with a better prognosis (HR = 0.25, 95% CI: 0.09–0.74, *p* = 0.004) [22]. Among the 11 studies that compared outcomes based on the use of ICI, seven reported improved survival with ICI therapy [23]. However, in one study analyzing the prognosis of 100 patients with SNMM, comparing periods before and after using ICI, no significant difference in survival was observed [24]. A 2023 single-arm trial evaluating combined nivolumab and ipilimumab postoperatively was reported, in which SNMM accounting for ~30% of cases demonstrated favorable outcomes, with a 1-year OS of 87% and a 2-year OS of 68% [25]. Grade ≥3 irAEs included diarrhea (14%), hypertension (14%), and hyponatremia (11%), suggesting a potentially high frequency of irAEs.

Although ICIs have been used primarily as adjuvant therapies, several reports suggest a role in first-line treatment [26,27]. ICI as neoadjuvant therapy in 36 patients with mucosal melanoma, including head and neck sites, had an ORR of 47% and a 3-year OS rate of 55% [26], with 8% achieving a complete response and avoided surgery. In our study, Case 10 received nivolumab prior to RT and remains without progression.

The occurrence of irAE is known to be associated with a favorable prognosis, as has been reported for various cancer types [28,29,30]. In our study, irAE occurred in four of 11 patients who received nivolumab, necessitating its discontinuation. However, three of the four patients maintained their response without recurrence after nivolumab was discontinued. Previous reports have shown an incidence of irAE of 60–61%, with 30–43% of cases necessitating the suspension or discontinuation of ICI therapy [23,24]. In several cancer types, including cutaneous melanoma, it is known that a subset of patients maintain a sustained therapeutic response even after permanent discontinuation of ICIs, and similar cases were identified in the present study [31,32].

This study has several limitations. First, this was a retrospective study, with only 11 patients receiving nivolumab. In addition, it included a mixture of cases in which nivolumab was administered as postoperative adjuvant therapy and cases in which nivolumab was administered for unresectable recurrence. Furthermore, various treatment modalities such as surgery, radiotherapy, and chemotherapy were used in combination or sequentially. In many ways, then, the two groups are far from comparable. Therefore, it is not possible to isolate clearly the specific contribution of immunotherapy to the observed outcomes, and the “statistically significant” findings we report should be interpreted with considerable caution. The finding that nivolumab was helpful in recurrent disease is noteworthy, and suggests that our earlier report suggesting its utility may be sound. Given the rarity of SNMM, prospective studies with clearly defined patient selection criteria are likely not plausible for it. Hence, continued accumulation of reported cases, with meta-analyses from time to time, should gradually build support for a role for ICIs in SNMM.

## 5. Conclusions

Nivolumab demonstrated favorable survival outcomes in patients with SNMM, and some patients maintained durable responses even after treatment discontinuation due to irAE. Although the total number of cases reported in meta-analyses is small, and data (including ours) is heterogeneous, our findings support and add to the literature supporting the use of ICIs in SNMM.

## Figures and Tables

**Table 1:** Details of treatment in all cases.

Case	Sex	Age	Stage	NAT	Treatment	Postoperative Treatment	AT	irAE	Recurrence	Treatment for Recurrence	Outcome
1	F	54	II	DAV	RT				+(lung, liver)	BSC	death
2	F	82	II		S	RT			+(unknown)	BSC	death
3	F	61	II	DAV	RT				+(bone)	BSC	death
4	F	51	II	DAV	RT				+(lung)	Nivo	death
5	M	57	I	DAV	S				+(lung)	DAV	death
6	F	68	II	DAV	RT				+(lung, bone, liver)	Nivo	death
7	F	79	IV		BSC				-		death
8	F	33	I		Nivo			Myasthenia gravis	+(lung, bone)	Pembro	death
9	F	69	I		S				+(local)	S → Nivo	death
10	M	80	II	Nivo	RT	Nivo			-		alive
11	M	67	III		S	RT			+(lung)	Nivo	death
12	M	78	I		S	RT			+(lung)	Nivo	death
13	F	71	II		S	RT	Nivo	Colitis Hypothyroidism	-		alive
14	M	73	II		S	RT	Nivo		+(lung)	Nivo + Ipi	alive
15	M	82	I		S	RT	Nivo	Skin disorders	-		alive
16	M	78	I		S	RT	Nivo	Skin disorders	-		alive

NAT—neoadjuvant therapy; DAV—dacarbazine and nimustine and vincristine; RT—radiation therapy; S—surgery; BSC—best supportive care; AT—adjuvant chemotherapy; irAE—immune-related adverse event.

## Data Availability

The data supporting the findings of this study are not publicly available due to ethical and privacy restrictions, but are available from the corresponding author upon reasonable request.
